# Sulfation of glycosaminoglycans depends on the catalytic activity of lithium-inhibited phosphatase BPNT2 *in vitro*

**DOI:** 10.1016/j.jbc.2021.101293

**Published:** 2021-10-08

**Authors:** Brynna S. Eisele, Zigmund Luka, Alice J. Wu, Fei Yang, Andrew T. Hale, John D. York

**Affiliations:** 1 Department of Biochemistry, Vanderbilt University, Nashville, Tennessee, USA; 2 Department of Pharmacology, Vanderbilt University, Nashville, Tennessee, USA

**Keywords:** bisphosphate nucleotidase 2 (BPNT2), chondrogenesis, chondroitin sulfate, enzyme catalysis, extracellular matrix, fibroblast, glycosaminoglycan, Golgi, molecular biology, N-linked glycosylation, C0S, unsulfated chondroitin, C4S, chondroitin-4-sulfate, C6S, chondroitin-6-sulfate, BPNT1, bisphosphate nucleotidase 1, BPNT2, bisphosphate nucleotidase 2, DMMB, dimethylmethylene blue, EV, empty vector, GAG, glycosaminoglycan, LiCl, lithium chloride, MEFs, mouse embryonic fibroblasts, NaCl, sodium chloride, PAP, 3′-phosphoadenosine-5′-phosphate, PAPS, phosphoadenosine-phosphosulfate

## Abstract

Golgi-resident bisphosphate nucleotidase 2 (BPNT2) is a member of a family of magnesium-dependent, lithium-inhibited phosphatases that share a three-dimensional structural motif that directly coordinates metal binding to effect phosphate hydrolysis. BPNT2 catalyzes the breakdown of 3′-phosphoadenosine-5′-phosphate, a by-product of glycosaminoglycan (GAG) sulfation. KO of BPNT2 in mice leads to skeletal abnormalities because of impaired GAG sulfation, especially chondroitin-4-sulfation, which is critical for proper extracellular matrix development. Mutations in *BPNT2* have also been found to underlie a chondrodysplastic disorder in humans. The precise mechanism by which the loss of BPNT2 impairs sulfation remains unclear. Here, we used mouse embryonic fibroblasts (MEFs) to test the hypothesis that the catalytic activity of BPNT2 is required for GAG sulfation *in vitro*. We show that a catalytic-dead *Bpnt2* construct (D108A) does not rescue impairments in intracellular or secreted sulfated GAGs, including decreased chondroitin-4-sulfate, present in *Bpnt2*-KO MEFs. We also demonstrate that missense mutations in *Bpnt2* adjacent to the catalytic site, which are known to cause chondrodysplasia in humans, recapitulate defects in overall GAG sulfation and chondroitin-4-sulfation in MEF cultures. We further show that treatment of MEFs with lithium (a common psychotropic medication) inhibits GAG sulfation and that this effect depends on the presence of BPNT2. Taken together, this work demonstrates that the catalytic activity of an enzyme potently inhibited by lithium can modulate GAG sulfation and therefore extracellular matrix composition, revealing new insights into lithium pharmacology.

Sulfation is a ubiquitous biological process in eukaryotes wherein a sulfate group from phosphoadenosine-phosphosulfate (PAPS, the universal sulfate donor) is transferred to a target substrate by sulfotransferase enzymes. This reaction yields the by-product 3′-phosphoadenosine-5′-phosphate (PAP), which is further catabolized to 5′-AMP by the bisphosphate nucleotidases (BPNT1 and BPNT2) (1, 2, 3, 4, 5). BPNT1 is localized to the cytoplasm, where sulfation of small molecules (such as hormones and xenobiotics) occurs (2). BPNT2 (previously known as LPM, IMPAD1, and gPAPP) is localized to the Golgi, which is the site of glycosaminoglycan (GAG) sulfation (1). Sulfated GAGs are important components of the extracellular matrix that serve important structural roles and facilitate cell-to-cell signaling (6).

A role for BPNT2 in regulating GAG sulfation was discovered through the generation of *Bpnt2*-KO mice. These mice die in the perinatal period, but pups have shortened limbs indicative of chondrodysplasia (1, 7). Further analysis of tissue from *Bpnt2*-KO pups on embryonic day 18.5 identified a significant decrease in GAG sulfation, particularly of chondroitin-4-sulfate (C4S) (1), which is necessary for the production of the cartilage matrix that precedes endochondral ossification of long bones. Subsequent studies by other groups identified an autosomal recessive human disorder caused by mutations in *BPNT2* and characterized by chondrodysplasia (8, 9, 10), reminiscent of the KO mouse phenotype, as well as other disorders of GAG sulfation (11). However, the mechanism by which loss or mutation of *Bpnt2* impairs GAG sulfation is not currently known.

The BPNT enzymes are members of a family of magnesium-dependent, lithium-inhibited phosphatases (1, 12). The precise mechanism and location of lithium-mediated inhibition of these enzymes were recently reported, establishing this family of enzymes as the direct targets of lithium (13). Lithium has been used for more than a half-century as a treatment for psychiatric disorders (14, 15), but its therapeutic mechanism remains unclear (16). Prior work suggests that lithium may modulate chondroitin sulfate (17, 18), but the mechanisms by which this could occur remain unknown. BPNT2 is a known modulator of chondroitin sulfation and a direct target of lithium (1). Thus, BPNT2 inhibition is a candidate mechanism for lithium's purported effects on chondroitin sulfate, and this inhibition may contribute to the therapeutic consequences or side effects of lithium treatment. However, previous studies have not established whether the consequences of *Bpnt2*-KO originate from a loss of BPNT2's catalytic activity (namely, the conversion of PAP to 5′-AMP) or another noncatalytic function. As more becomes known about nonenzymatic roles of proteins and the genes encoding them, including structural functions (*e.g.*, formation of protein complexes and substrate channeling (19)) and signaling functions (*e.g.*, genes containing noncoding microRNAs that regulate pertinent pathways (20)), we cannot assume that a phenotypic consequence of knocking out a gene is necessarily due to loss of a catalytic function.

The objective of this study was to investigate whether the loss of BPNT2's catalytic function underlies the chondrodysplastic phenotype observed in mice and humans. We established a model system to study BPNT2's effects on GAG sulfation *in vitro* using embryonic fibroblasts cultured from *Bpnt2*-KO mice. We then used genetic complementation to examine the impact of mutations in *Bpnt2* on GAG sulfation. We studied 3 *Bpnt2* mutations, which happen to be in close proximity to the active site/metal-binding domain (one that ablates catalytic activity, two that are known to cause chondrodysplasia in humans (8)). We demonstrate herein that these mutations impair GAG sulfation. We further show that the treatment of mouse embryonic fibroblast (MEF) cultures with lithium chloride (LiCl) decreases GAG sulfation; these effects are dependent on the presence of BPNT2, consistent with BPNT2 being an *in vivo* target of the drug.

## Results

### Generation of an *in vitro* model system to analyze GAG sulfation

**Figure 1:**
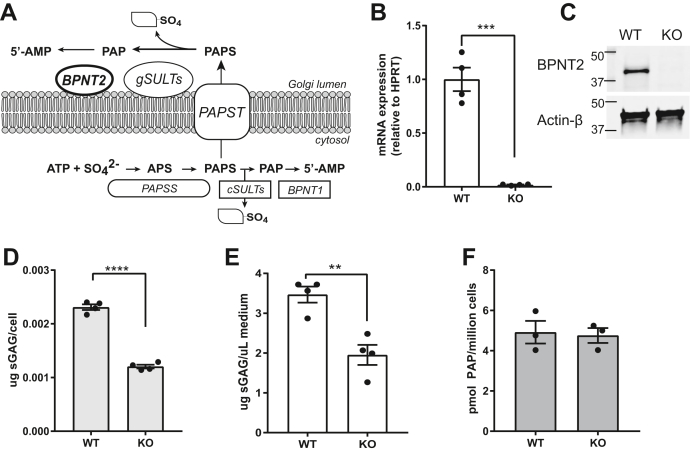
**Loss of Bpnt2 impairs glycosaminoglycan sulfation but does not alter the PAP level.***A*, illustration of intracellular sulfation pathways, wherein BPNT2 hydrolyzes PAP, a by-product of sulfation, to AMP. *B*, absent expression of Bpnt2 mRNA (∗∗∗*p* = 0.0001) in Bpnt2-KO MEFs as determined by quantitative PCR and (*C*) absent BPNT2 protein in Bpnt2-KO MEFs, as determined by Western blot. *D*, Bpnt2-KO MEFs exhibit decreased levels of both intracellular (*left*, ∗∗∗∗*p* < 0.0001) and (*E*) secreted (*right*, ∗∗*p* = 0.0034) sulfated glycosaminoglycans, as determined by the DMMB assay. *F*, Bpnt2-KO MEFs do not show changes in the PAP level relative to WT cells. The bars show the mean ± SEM. Significance analyses are the results of unpaired Student's *t* test (two-sided). APS, adenosine phosphosulfate; BPNT1, bisphosphate nucleotidase 1; BPNT2, bisphosphate nucleotidase 2; cSULTs, cytosolic sulfotransferases; DMMB, dimethylmethylene blue; gSULTs, Golgi-resident sulfotransferases; MEFs, mouse embryonic fibroblasts; PAP, 3′-phosphoadenosine-5′-phosphate; PAPS, phosphoadenosine-phosphosulfate; PAPSS, PAPS synthase; PAPST, PAPS transporter.

BPNT2 is a Golgi-resident protein that has a demonstrated role in Golgi-localized sulfation reactions. The major components of this sulfation pathway are illustrated in Figure 1*A*. Loss of BPNT2 is known to impair the upstream sulfation of GAGs, but previous studies have not established whether this effect stems from the loss of BPNT2 catalytic activity or another noncatalytic function. To investigate this mechanism, we first sought to generate an immortalized cell system to study the function of BPNT2 with respect to alterations in GAG sulfation. We elected to use embryonic fibroblasts because (1) these cells were easily attainable from the *Bpnt2*-KO mouse line developed by our laboratory (Jackson, #012922), (2) MEFs derive from mesenchyme, which give rise to connective tissues that are primarily responsible for GAG synthesis *in vivo*, and (3) MEFs can be readily immortalized to facilitate genetic manipulations and prolonged study. MEFs were harvested on embryonic day 12.5 of pregnancies resulting from *Bpnt2*-heterozygous crosses. *Bpnt2*-WT and *Bpnt2*-KO MEFs obtained from littermates were then immortalized by lentiviral expression of SV40T antigens. Absence of BPNT2 in these lines was further confirmed by analyzing mRNA expression using quantitative PCR (Fig. 1*B*) and by immunoblotting for BPNT2 protein (Fig. 1*C*). For our analyses, the most relevant property of the cells was their ability to synthesize and secrete sulfated GAGs, the most abundant of which is chondroitin sulfate. The cells primarily responsible for chondroitin sulfate production *in vivo* are chondrocytes, but chondrocytes are not well suited to long-term culture involving repeated passaging. However, chondrocytic properties can be induced in cells of mesenchymal origin by maximizing cell–cell contact in cultures and supplementing media with certain growth factors (21). This method of culturing immortalized MEFs allowed us to investigate the effects of BPNT2 on GAG sulfation *in vitro*, without having to repeatedly harvest primary cells, and manipulate gene expression to generate stable cell lines expressing mutant versions of BPNT2. *Bpnt2*-KO MEFs display decreased total GAG sulfation as measured by the dimethylmethylene blue (DMMB) assay (Fig. 1*D*), normalized to the cell number. KO MEFs also secrete fewer sulfated GAGs into the culture medium (Fig. 1*E*). We were also interested in whether these cells displayed measurable alterations in the PAP level, which might be expected in the absence of BPNT2, but we did not detect any difference in PAP between WT and KO cells (Fig. 1*F*). Nonetheless, the alterations in sulfation in the immortalized MEFs recapitulate impairments in sulfation seen in *Bpnt2*-KO mice. We therefore deemed this an appropriate model for our investigations.

### Bpnt2 mutations that cause chondrodysplasia are located near the metal-binding/catalytic domain

**Figure 2:**
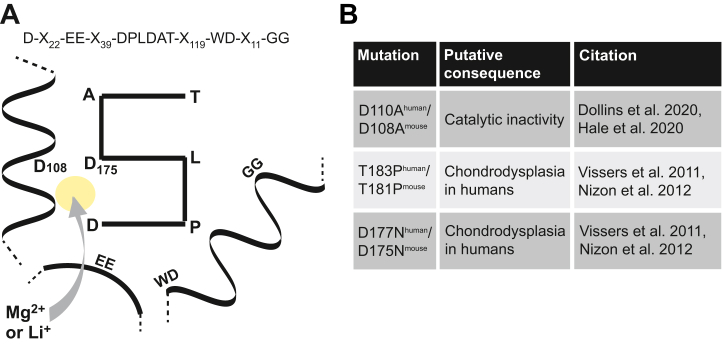
**Summary of *Bpnt2* mutations in relation to the metal-binding/catalytic domain.***A*, graphical simplification of metal-binding/catalytic domain defining magnesium-dependent/lithium-inhibited phosphatases; numbered amino acids are those in murine *Bpnt2*; *yellow circle* represents the metal-binding pocket, where magnesium binds under active conditions and lithium binds under inhibitory conditions. *B*, table showing mutations investigated herein, which are in close proximity to the metal-binding structural motif. BPNT2, bisphosphate nucleotidase 2.

Murine BPNT2's three-dimensional core structural motif (which defines the family of lithium-inhibited phosphatases) has been simplified and represented graphically in Figure 2*A*. This region of BPNT2 is highly conserved across species (8). Three aspartic acid (D) residues provide a negatively charged environment conducive to the binding of positively charged metal cations: divalent magnesium is a necessary cofactor for phosphate hydrolysis, whereas monovalent lithium inhibits this hydrolysis (13). Mutation of the first aspartic acid that composes this pocket (D110^human^/D108^mouse^) to alanine renders the family members catalytically inactive (13, 22). Interestingly, two missense mutations in *Bpnt2* localized near this locus are known to cause chondrodysplasia in humans (8, 9). A summary of these mutations is shown in Figure 2*B*. These mutant versions of *Bpnt2* have previously been predicted to have effects on the enzymatic activity because of localization near the presumed active site, based on structural comparisons to BPNT1 (PDB ID: 2WEF) (8), as no structure has yet been determined for BPNT2. Indeed, one of these mutations, D177N^human^/D175N^mouse^, is in another of the 3 aspartic acid residues that compose the negatively charged pocket, while T183P^human^/T181P^mouse^ is located just six amino acids further downstream.

### Expression of Bpnt2 mutants revealed a novel N-glycosylation locus on chondrodysplasia-associated mutant Bpnt2^D175N^

**Figure 3:**
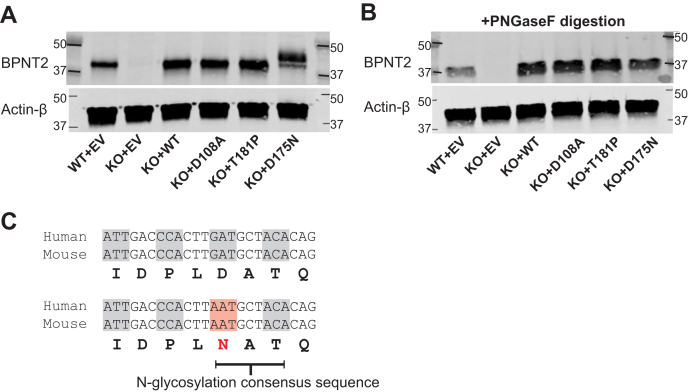
**Generation of mutant *Bpnt2* MEF lines.***A*, blot for BPNT2 and actin on protein extracted from *Bpnt2* MEF lines; *arrows* in D175N lane denote 2 bands, representing singly and doubly glycosylated protein. *B*, blot of proteins extracted from MEF lines, treated with PNGaseF to remove N-glycosyl groups; PNGaseF removes both glycosyl groups, resulting in a single band for BPNT2-D175N. Note that all bands shift downward with PNGaseF, as native murine BPNT2 has one N-glycosylation site at N257. *C*, a selection of *Bpnt2* sequence from mouse and human *Bpnt2*. In both, the D175N/D177N mutation results in the generation of an N-glycosyl consensus sequence. BPNT2, bisphosphate nucleotidase 2; MEF, mouse embryonic fibroblast.

We next used site-directed mutagenesis and a retroviral expression system to generate MEF lines that exclusively express mutant forms of murine *Bpnt2*: *Bpnt2*^D108A^, *Bpnt2*^T181P^, and *Bpnt2*^D175N^. We also generated a WT *Bpnt2*-complemented cell line (KO + WT) as a control and WT and KO lines transduced with an empty vector (EV) control retrovirus. Success of viral transduction was determined by Western blotting for BPNT2 (Fig. 3*A*). Surprisingly, BPNT2^D175N^ mutant protein appeared to be partially shifted upward, displaying a second band of higher molecular weight relative to other BPNT2 isoforms. Native BPNT2 is localized to the Golgi, and like other membrane-associated proteins, it contains an N-glycosylation consensus sequence (N-X-S/T (23)) at N259^human^/N257^mouse^. The novel asparagine in BPNT2^D175N^ made us inquire as to whether an additional N-glycosylation locus was generated by this mutation, as a secondary glycosylation event could explain the increased molecular weight of the detected protein. Figure 3*B* shows a section of cDNA for both human and mouse *Bpnt2*. In both the human and mouse, the mutation of this aspartic acid to asparagine results in the generation of an N-glycosylation consensus sequence: N-A-T. To test whether the heavier band was indeed due to an additional N-glycosyl modification, protein extracts from cell lines were treated with PNGaseF. Native BPNT2's one glycosylation site at N259^human^/N257^mouse^ is cleaved with PNGaseF treatment, resulting in a downward shift of the protein. Treatment of BPNT2^D175N^ with PNGaseF cleaves both N-glycosyl groups, eliminating the double band and producing a protein of the same size as those seen in all other PNGaseF-treated cell lines (Fig. 3*C*). The generation of this additional N-glycosylation site has not been previously described in association with this mutation.

### WT Bpnt2 rescues impairments in overall sulfated GAGs, whereas mutant Bpnt2 constructs do not

**Figure 4:**
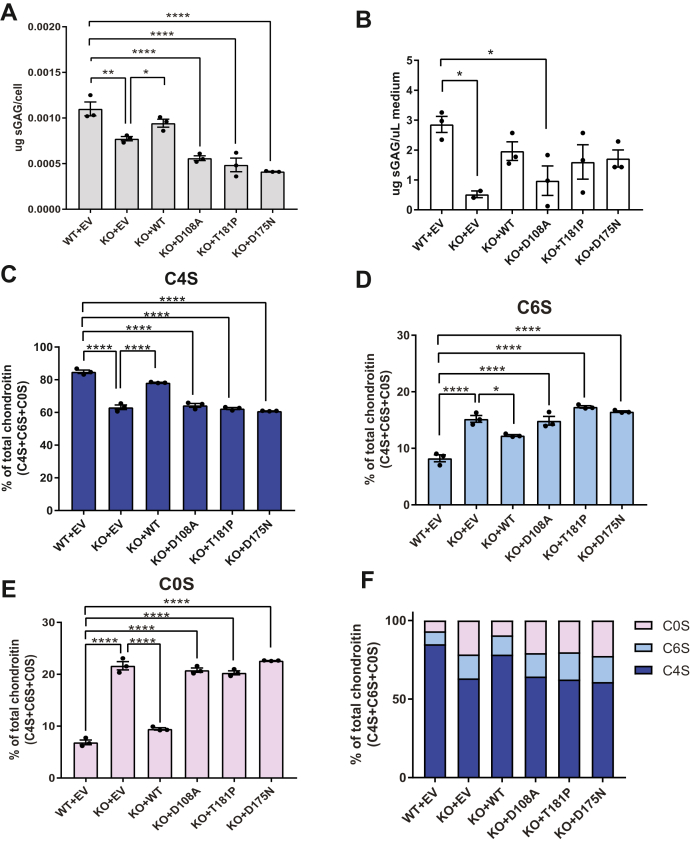
**MEFs expressing mutated Bpnt2 exhibit decreased sulfated glycosaminoglycans, including decreased chondroitin-4-sulfate.***A*, sulfated GAG levels in Bpnt2-mutant cells, as measured by the DMMB assay. *B*, sulfated GAG levels in the medium of Bpnt2-mutant cell cultures, as measured by the DMMB assay. Alterations in (*C*) chondroitin-4-sulfate, (*D*) chondroitin-6-sulfate, and (*E*) unsulfated chondroitin levels in Bpnt2-mutant cell cultures, as measured by chondroitin disaccharide HPLC. *F*, summary of alterations in chondroitin-sulfation profile across Bpnt2-mutant lines. The error bars show the mean ± SEM. Denoted significance indicates the results of Tukey's post hoc tests after significant one-way ANOVA. ∗*p* < 0.05, ∗∗*p* < 0.01, ∗∗∗∗*p* < 0.0001. BPNT2, bisphosphate nucleotidase 2; C0S, unsulfated chondroitin; C4S, chondroitin-4-sulfate; C6S, chondroitin-6-sulfate; DMMB, dimethylmethylene blue; EV, empty vector; GAG, glycosaminoglycan; MEF, mouse embryonic fibroblast.

We next sought to examine the consequences of these mutations on GAG sulfation. To facilitate the synthesis of GAGs and extracellular matrix components, we cultured three-dimensional cell pellets for each MEF line over a period of 7 to 14 days. Media were collected from pellets immediately before harvest. The total sulfated GAG from cell pellets was quantified using a colorimetric DMMB assay and then normalized to cell count. These results are depicted in Figure 4*A*. We observed a significant decrease in sulfated GAG in the KO line, which was rescued by expressing WT *Bpnt2*. In contrast, expression of *Bpnt2*^D108A^ did not rescue this decrease, nor did *Bpnt2*^T181P^ or *Bpnt2*^D175N^. The level of secreted sulfated GAGs in the media (collected at pellet harvest) was also measured and is shown in Figure 4*B*. Again, we observed a significant decrease in secreted/extracellular sulfated GAGs in the KO line, which was not rescued with expression of *Bpnt2*^D108A^, but did appear to be rescued with expression of other *Bpnt2* mutants.

### Mutant Bpnt2 constructs do not rescue specific alterations in chondroitin sulfation

We next used HPLC to evaluate specific alterations in chondroitin sulfation. Isolated GAGs can be digested by chondroitinase enzymes to yield individual sulfated chondroitin disaccharides. These disaccharides can then be fluorescently labeled and resolved by HPLC to identify specific sulfation moieties. Previous work has demonstrated that *Bpnt2*-KO mouse embryos exhibit impairments in the overall levels of 4-sulfated disaccharide (Δdi-4S, or C4S) that correspond with increased levels of unsulfated chondroitin (Δdi-0S or C0S) (1). We observed a decrease in the ratio of C4S to total chondroitin disaccharides (C4S + C6S + C0S) in KO + EV MEFs relative to WT + EV MEFs, which was in large part rescued by complementing WT *Bpnt2* back into the line (KO + WT). However, this decrease was not rescued by the expression of catalytic-dead *Bpnt2* or chondrodysplasia-associated mutant *Bpnt2* (Fig. 4*C*). Interestingly, we observed a correspondent increase in the ratio of chondroitin-6-sulfate (Δdi-6S or C6S) in KO cells, which was partially restored with WT complementation and which remained elevated in mutant *Bpnt2* lines (Fig. 4*D*). The increase in C6S has not been previously reported in association with *Bpnt2*-KO cells. However, overall ratios of C0S are increased in KO cells, restored to near-WT levels with back complementation, and significantly elevated in mutant *Bpnt2* lines (Fig. 4*E*). These changes are summarized in Figure 4*F*.

### Lithium decreases intracellular and extracellular sulfated GAGs, including C4S

**Figure 5:**
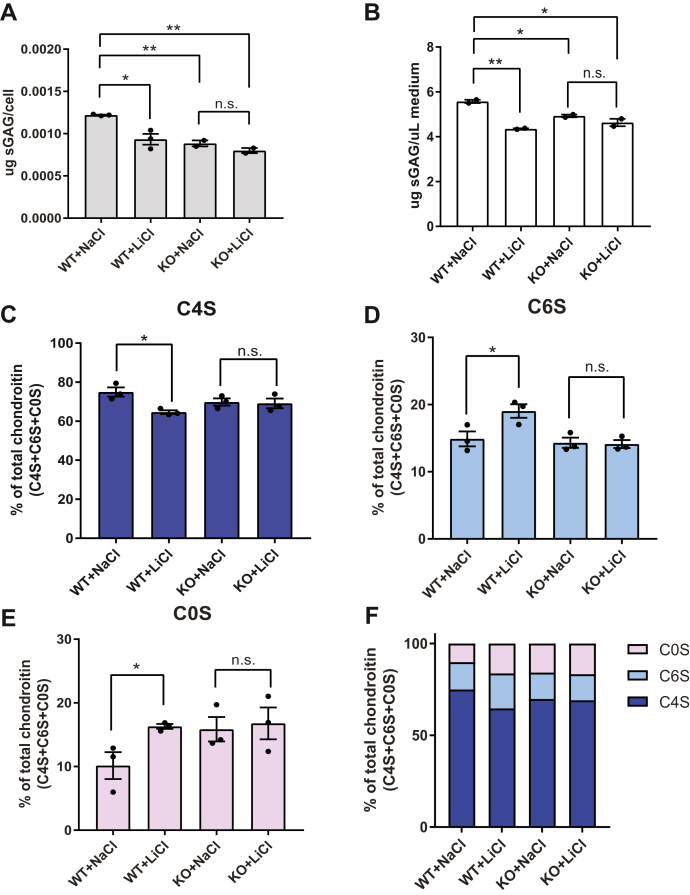
**Lithium treatment decreases overall GAG sulfation, including chondroitin-4-sulfation, in WT cells, but not in Bpnt2-KO cells.***A*, sulfated GAG analyses performed on cells treated with 10 mM NaCl or LiCl. *B*, sulfated GAG analyses performed on the culture medium of cells treated with 10 mM NaCl or LiCl. Alterations in (*C*) chondroitin-4-sulfate, (*D*) chondroitin-6-sulfate, and (*E*) unsulfated chondroitin levels in treatment groups, as measured by chondroitin disaccharide HPLC. *F*, summary of alterations in chondroitin-sulfation profile across treatment groups. The error bars show the mean ± SEM. Denoted significance indicates the results of two-sided Student's *t* test. ∗*p* < 0.05, ∗∗*p* < 0.01. BPNT2, bisphosphate nucleotidase 2; C0S, unsulfated chondroitin; C4S, chondroitin-4-sulfate; C6S, chondroitin-6-sulfate; GAG, glycosaminoglycan; LiCl, lithium chloride; NaCl, sodium chloride; n.s., not significant.

The discovery that the loss of BPNT2 catalytic function underlies impairments in overall GAG sulfation is particularly relevant because BPNT2 is potently inhibited by the psychopharmacologic agent lithium (1). To test whether lithium impairs total GAG sulfation, we treated both WT and *Bpnt2*-KO MEFs with 10 mM LiCl, using an equal concentration of sodium chloride (NaCl) as a control. We observed a decrease in intracellular GAG sulfation in lithium-treated WT MEFs, as compared with sodium-treated WT MEFs (Fig. 5*A*). The loss of *Bpnt2* in cells treated with sodium resulted in a marked reduction in GAG sulfation similar to WT MEFs treated with lithium (Fig. 5*A*). Importantly, treatment of *Bpnt2*-KO MEFs with lithium did not further reduce sulfation of GAGs (Fig. 5*A*). Likewise, we observed a significant reduction in the sulfation of GAGs secreted into the culture medium in lithium-treated as compared with control sodium-treated samples (Fig. 5*B*). Again, *Bpnt2*-KO MEFs treated with LiCl did not exhibit any additional decrease in secreted sulfated GAGs.

We next used HPLC to resolve chondroitin disaccharides in WT and *Bpnt2*-KO cells treated with lithium, and we observed a significant decrease in C4S (Fig. 5*C*) in LiCl-treated WT cells alongside significant increases in C6S (Fig. 5*D*) and C0S (Fig. 5*E*). *Bpnt2*-KO cells did not exhibit these alterations when treated with LiCl, demonstrating that lithium does not have any additional effects on chondroitin sulfation patterns in cells that lack *Bpnt2*. Collectively, the similarity between sulfation patterns in LiCl-treated cells and *Bpnt2*-KO cells is appreciated in Figure 5*F* and is consistent with a role for *Bpnt2* in mediating lithium's effect on GAG sulfation. As a follow-up to these experiments, we evaluated whether lithium treatment altered the PAP level in WT or *Bpnt2*-KO MEFs, and we did not observe significant alterations (Fig. S1), nor did we observe alterations in the expression of key members of the chondroitin sulfation pathway (Fig. S2).

## Discussion

In this work, we use an *in vitro* fibroblast model to demonstrate that KO of a lithium-inhibited enzyme, BPNT2, impairs overall GAG sulfation (especially chondroitin-4-sulfation), and that this impairment stems specifically from a loss of the catalytic activity of BPNT2, as a catalytic-dead construct does not rescue these impairments. We also evaluated two missense mutations in BPNT2 that are associated with chondrodysplasia in humans and are adjacent to the core catalytic motif. We therefore suspect that these mutations interfere with BPNT2 catalysis. Vissers *et al*. (8), who initially described these mutations, posited that the mutation of threonine 183 to proline in human *BPNT2* would produce a helix-breaker effect, which would alter secondary BPNT2 structure. They also suggested that the loss of the charged aspartic acid side chain in the D177N mutation would affect the binding of metal cations at the active site (8). In this work, we recapitulated impairments in chondrogenesis caused by these two missense mutations, and we further report that the D(177/175)N mutation generates an N-glycosylation consensus sequence in both human and mouse *Bpnt2*. N-glycosylation is a protein modification that occurs cotranslationally (24), which may affect protein folding. Additional kinetic and protein biochemical assays will be important in understanding precisely how these mutations influence BPNT2 catalysis.

As previously reported, we identified decreases in C4S and increases in C0S as a result of the loss of BPNT2, which we also attribute to the disrupted BPNT2 catalytic activity. Of note, we also identified increases in C6S, which were not previously identified in tissues (whole-embryo preparations) from *Bpnt2*-KO mice. Contrasting roles for C4S and C6S have been described, wherein C4S is more abundant in developing cartilage, whereas C6S is more abundant in mature and articular cartilage (25). In the nervous system, C6S is more abundant in the developing brain, where it promotes synaptic plasticity, whereas C4S is more abundant in the adult brain, where it limits plasticity and promotes synaptic stability (26, 27). Importantly, decreases in C6S have been identified in the brains of human patients with bipolar disorder (18), and lithium treatment is associated with increases in C6S (18), which is thought to be corrective. We now present evidence that inhibiting the catalytic activity of a molecular target of lithium can increase C6S levels in a mammalian cell model. The increase in C6S could be a result of shunting or flux, given the reductions in C4S and significant elevation of C0S. It is notable that GAG sulfation overall was still decreased in response to the loss of BPNT2 activity, as measured by the DMMB assay, which is not specific for any species of sulfated GAGs.

Although our results clearly indicate that BPNT2 activity is required for altering sulfated GAG/chondroitin sulfation, we are not able to distinguish exactly how this is accomplished. Is it through altering the production of 5′-AMP or through defects in the consumption of PAP? In our previous studies of BPNT2's cytosolic counterpart BPNT1, we found clear evidence of PAP accumulation in mutant animals and cells, which resulted in metabolic toxicity (4, 22). An accumulation of PAP with BPNT2-KO could feedback-inhibit sulfotransferases (28), impairing GAG sulfation. However, we did not observe an increase of PAP in *Bpnt2*-KO cells. It may be that PAP only needs to locally accumulate within the Golgi, where BPNT2 is located, to effectively inhibit Golgi-resident sulfotransferases. Meanwhile, BPNT1 is still metabolizing PAP in the cytosol. The relative proportion of PAP accumulation in the Golgi to the total amount of PAP in the cell may be too small for such a difference to be detectable by our assays. Isolating only the PAP that is located within the Golgi-lumen has thus far proven to be a technically complex undertaking, as traditional subcellular fractionation studies by our group have not yielded measurable PAP levels in the Golgi-associated fractions. If, on the other hand, the phenotypes observed in BPNT2 mutants are due to the failure to produce 5′-AMP, then it is possible that PAPS transport into the Golgi is impaired. Studies of the PAPS transporter, which moves the sulfate donor PAPS from the cytosol into the Golgi, indicate it is a member of the antiporter family of proteins that may exchange PAPS for 5′-AMP (29, 30). The diminished production of 5′-AMP in the Golgi resulting from the loss of BPNT2 activity could prevent PAPS from entering the Golgi and being used for sulfation. Notably, we did not observe alterations in the expression of the PAPS transporters or chondroitin sulfotransferases in WT or KO cells treated with lithium (Figure S2), but there are other mechanisms by which the functions of these proteins could be altered. Further work will be required to determine which, if any, of these mechanisms underlies the observed sulfation impairments.

In this work, we also show that treatment of MEFs with 10 mM LiCl alters sulfation, and that this alteration is dependent on the presence of BPNT2. Prior work suggests that lithium negatively regulates chondroitin sulfate proteoglycans that would otherwise prevent axon regeneration after spinal cord injury (17). It has also been shown that lithium promotes neurite outgrowth (31, 32), a process which is normally restricted by chondroitin sulfate (33). Consistent with these prior findings, we identified decreases in the sulfated GAG when MEFs were treated with lithium. *Bpnt2*-KO MEFs displayed impaired GAG sulfation in a manner that mimicked WT MEFs treated with lithium and did not exhibit additive decreases in sulfation with lithium treatment. When specifically analyzing chondroitin disaccharides, we identified alterations in the WT MEFs treated with lithium, consistent with the effects previously identified in *Bpnt2*-KO MEFs, but we did not identify additional alterations when *Bpnt2*-KO MEFs were treated with lithium. These data suggest that lithium's effects on chondroitin sulfation are mediated by BPNT2.

The field of psychopharmacology has yet to reach a consensus on how lithium remains so effective in treating bipolar disorder despite a vast array of other recent psychopharmacologic advances. Although this work does not decisively establish lithium's mechanism of action, it does present evidence that loss of the catalytic activity of known target of lithium, BPNT2, mediates decreases in the sulfated GAG, and observable lithium-mediated decreases in sulfation may be due to the loss of BPNT2. On the whole, this work provides a basis for an interesting new hypothesis into how lithium could elicit its psychiatric effects.

## Experimental procedures

### MEF harvesting and culture methods

Cells were obtained from the *Bpnt2*-KO mouse line previously generated by our laboratory and available through Jackson Laboratory mouse repository (Jackson, #012922). Details regarding the development of this mouse line can be found in the study by Frederick *et al*. (1). MEFs were obtained from embryonic day 12.5 pups from heterozygous (*Bpnt2*+/-) breeding pairs. Cells from each pup were genotyped (according to Frederick *et al*. (1)) and cultured in Dulbecco's modified Eagle's medium (DMEM) + 4.5 mg/dl glucose and L-glutamine (Gibco), with 10% fetal bovine serum and 1% penicillin/streptomycin (basal medium). The cells were incubated at 37 °C with 5% CO_2_ for the duration of culture. The cells were immortalized by lentiviral expression of SV40 large and small T antigens—briefly, SV40T antigen lentiviral plasmid (Addgene, #22298) was packaged in HEK 293T cells using helper plasmids pMD2.G (Addgene, #12259) and psPAX2 (Addgene, #12260). Forty-eight hours after transfection of all three plasmids, 3 ml of cell media was collected, filtered through a 0.45-um sterile filter, and added directly to the MEF culture medium containing 8 μg/ml polybrene. For all studies of mouse-derived cell lines, protocols were performed under strict adherence to approved protocols obtained through IACUC review boards at Duke and Vanderbilt Universities (for the laboratory of J. D. Y.).

### Promotion of chondrogenesis in MEFs

To enhance the production of GAGs while in three-dimensional culture, the basal medium was changed to the chondrogenic medium: DMEM + 4.5 mg/dl glucose and L-glutamine (Gibco), with 10% fetal bovine serum, 1% penicillin/streptomycin, 1% ITS + supplement (Corning), 0.1 μM dexamethasone, 200 μM ascorbic acid, and 10 ng/ml TGF-β1 (Peprotech). For chondrogenic three-dimensional pellet culture, approximately one million cells were seeded in sterile screw-cap 1.7-ml conical tubes and centrifuged at 500*g* for 5 min to form pellets. The cells were cultured as pellets in these tubes with the caps loosened. Pellet media (700 μl per tube) were changed 2 times weekly until cells were harvested for downstream analysis, 7 to 14 days after seeding. For lithium experiments, the medium contained 10 mM LiCl or NaCl for the duration of pellet culture.

### Generation of mutant Bpnt2 MEF lines

Mouse *Bpnt2* cDNA was cloned into the pBABE-puro (Addgene, #1764) retroviral vector using BamHI and SalI restriction sites. The plasmid was subsequently mutagenized using traditional site-directed mutagenesis methods to generate D108A, T181P, and D175N mutants, and mutagenesis was verified by Sanger sequencing. Retroviral vectors were each cotransfected with VSV.G (Addgene, #14888) and gag/pol (Addgene, #14887) vectors into HEK 293T cells using GenJet DNA transfection reagent (SignaGen). Two milliliter of the viral supernatant was harvested 48 h after transfection, filtered to remove cells, and added directly to separate *Bpnt2*-KO MEF cultures containing 8 μg/ml polybrene (Invitrogen). Approximately 24 h after viral transduction, 3 μg/ml puromycin was added to kill nontransduced cells. The cells were incubated in puromycin media for 3 days before being passaged into media without puromycin. Efficacy of transduction was confirmed by measuring *Bpnt2* mRNA and protein expression.

### Quantitative PCR

RNA was collected from the culture samples using the Qiagen RNeasy Mini Kit, including treatment with Qiagen DNase I. cDNA was synthesized from 1 μg of RNA using the iScript cDNA Synthesis Kit from Bio-Rad. PCR was carried out using SsoAdvanced Universal SYBR Green Supermix (Bio-Rad) according to the manufacturer instructions. *Bpnt2* mRNA expression was normalized to HPRT mRNA expression to determine relative transcript enrichment. Primer sequences can be found in Table S1.

### Immunoblotting

Protein was collected from the cells lysed in RIPA buffer with the protease inhibitor (Roche). Protein extracts were passed through a 25g needle to break up DNA and subsequently quantified using the BCA assay. Ten microgram of total protein was loaded per lane onto a 12% SDS-PAGE gel (Bio-Rad), which was run at 100 V for 2 h. The proteins were transferred to a 0.2-μm PVDF membrane (Bio-Rad) using Trans-Blot Turbo (Bio-Rad) at 1.3 A for 7 min. Blots were incubated in primary antibodies (sheep anti-BPNT2, 1:1000, Invitrogen, #PA5-47893; mouse anti-Actin, 1:1000, Invitrogen, #MA1-744) overnight at 4 °C, washed 3 times in 0.1% TBS-Tween, then in secondary antibodies (AlexaFluor680 anti-sheep 1:20,000; AlexaFluor800 anti-mouse 1:20,000) for 2 h at room temperature, and washed 3 times in 0.1% TBS-T. The blots were imaged on LI-COR Odyssey.

### PNGaseF digestion

Ten microgram of the protein extract was digested with PNGaseF (NEB) according to the manufacturer instructions. The full digest products were subsequently run on gel as described above.

### Measurement of PAP in MEFs

Briefly, approximately one-million-cell MEF pellets that had been cultured as pellets for 14 days were boiled for 3 min in 150 μl of the PAP isolation buffer (50 mM glycine, pH 9.2) and disrupted mechanically with a tissue pestle. The homogenates were clarified by centrifugation at 16,100*g*, at 4 °C for 20 min. Then, 0.2 volume of chloroform was added, and samples were vortexed to mix. The samples were again centrifuged at 16,100*g*, at 4 °C for 20 min. The upper aqueous phase was then collected and used for the assay. To quantify PAP levels, we used a colorimetric microplate absorbance assay in which recombinant mouse SULT1A1-GST is used to transfer a sulfate group from p-nitrophenyl sulfate to 2-naphthol, using PAP as a catalytic cofactor (34). Briefly, 20 μl of the tissue lysate was incubated with 180 μl of the PAP reaction mixture (100 mM bis·Tris propane [pH 7.0], 2.5 mM β-mercaptoethanol, 2.5 mM p-nitrophenyl sulfate, 1 mM β-naphthol, and 1 μg of PAP-free recombinant mouse SULT1A1-GST). Reaction velocities were determined by monitoring the production of 4-nitrophenol at 405 nm. The concentrations of PAP in lysates were determined by comparing the reaction rates acquired from kinetic analysis to those of a series of PAP standards run concurrently on the same plate.

### DMMB assay

Media were collected from cell pellets upon harvest and kept at −20 °C until used for downstream analyses. After removing the media, cell pellets were rinsed in 1X PBS. The pellets were then incubated in 300 μl 10 mM Tris HCl (pH 7.5) solution containing 100 μg/ml proteinase K (Roche) at 60 °C overnight followed by 30 min at 90 °C to denature Proteinase K. Forty microliter of each sample was loaded onto a clear-bottom 96-well plate in duplicate, and 200 μl of the DMMB reagent, pH 1.5 (prepared according to Zheng and Levenston 2015 (35)) was added using a multichannel pipette. Absorption was immediately measured at 525 and 595 nm, and 595 measurement was subtracted from 525 to yield the final reading. The quantity of sulfated GAGs was determined by comparison with a standard curve of bovine C4S (Sigma) prepared in 10 mM Tris HCl. The amount of sulfated GAGs was normalized to the cell count across samples. For the analysis of secreted sulfated GAGs in media, 60-μl media was added directly to the clear-bottom 96-well plate in duplicate, and readings were compared against bovine C4S standards prepared in the culture medium.

### HPLC analysis of chondroitin-sulfate disaccharides

One-million-cell pellets were homogenized in 400-μl GAG preparation buffer (50 mM Tris, pH 8.0, 10 mM NaCl, and 3 mM MgCl_2_), containing 4 μl of 2 mg/ml Proteinase K, using tissue pestle. The sample was incubated overnight at 56 °C. After digest, the samples were heated at 90 °C for 30 min to denature Proteinase K. The precipitated material was separated by centrifugation. The sample buffer was changed to 0.1 M ammonium acetate, pH 7.0, by using 3-kDa Millipore concentrator by concentration/dilution until the initial concentration of the homogenization buffer decreased 500 times. The volume of concentrated samples was adjusted to 70 μl, and 3 μl of chondroitinase ABC (1.4 U/ml stock solution, containing BSA; Seikagaku) was added to each sample. The reaction mixture was incubated at 37 °C for 4 h. After chondroitinase ABC cleavage, 130 μl of water was added. The released disaccharides were filtered through a 10-kDa concentrator. This procedure was repeated once more to improve yield. GAG samples were lyophilized using a SpeedVac at 25 °C overnight. The lyophilized samples were stored at −80 °C until fluorescent labeling. The labeling of disaccharides was performed with 2-aminobenzamide by a published procedure (36). An aliquot of 5 to 7 μl of the labeling mixture (0.35 M 2-aminobenzamide and 1 M NaCNBH_3_ solution in 30% acetic acid in dimethyl sulfoxide) was added to the lyophilized samples or disaccharide standards, and the mixture was incubated for 3 h at 65 °C. The labeling reaction mixtures were spotted on a strip of Whatman 3MM Chr paper and washed with 1 ml of acetonitrile six times. Cleaned disaccharides were eluted with three aliquots of 50, 75, and 75 μl of water by using a 0.2-um centrifugal device. The analysis of labeled disaccharides was performed by HPLC. The HPLC system included Waters 515 Pumps, Waters 517 Plus Autosampler, Waters Pump Control Module II, and Shimadzu RF-10AXL spectrofluorometer detector under Waters Empower software. Sample analysis was performed on a SUPELCOSIL LC-NH_2_ (25 cm × 4.6 mm) (Sigma) column. The column was equilibrated with 16 mM NaH_2_PO_4_ with a flow rate of 1 ml/min. The samples of 50 to 100 μl were injected and eluted with 60-min linear gradient 16 mM–800 mM NaH_2_PO_4_ with a flow rate of 1 ml/min as in Yoshida *et al*. (37). The disaccharide elution was monitored by fluorescence at 420 nm with excitation at 330 nm. The peaks were identified by comparison with C4S, C6S, and C0S chondroitin standards (Sigma). Calculations were determined by integrating each peak on the resultant chromatogram and calculating ratios of chondroitin species. The chromatograms were analyzed by Empower software.

## Data availability

All relevant data are contained within the article. Additional information may be requested by contacting the corresponding author.

## Supporting information

This article contains supporting information.

## Conflict of interest

The authors declare that they have no conflicts of interest with the contents of this article.
