# Supplementary material for: Sulfation of glycosaminoglycans depends on the catalytic activity of lithium-inhibited phosphatase BPNT2 in vitro
Source: J Biol Chem. 2021 Oct 8;297(5):101293. doi: 10.1016/j.jbc.2021.101293 (PMC8551643; doi:10.1016/j.jbc.2021.101293)
Supplement: Figures S1 and S2 and Table S1 [file mmc1.pdf]

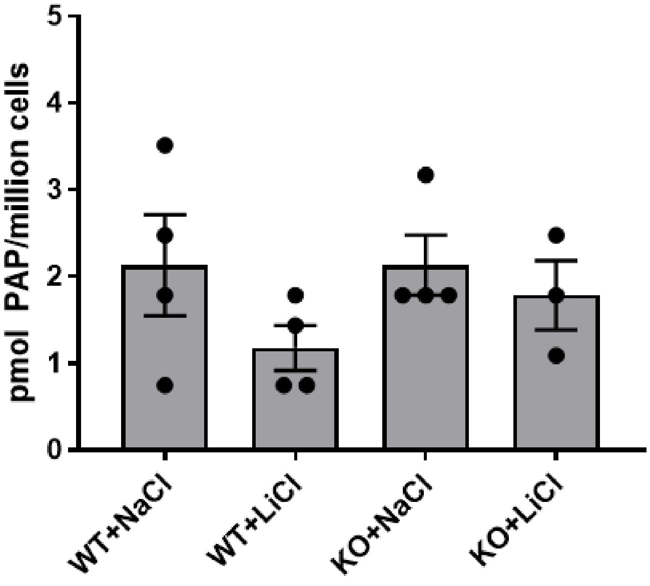

Figure S-1. Lithium treatment does not alter PAP level in WT or BPNT2-KO MEFs. Cells were treated with 10mM LiCl or 10mM NaCl and cultured as 3-dimensional cell pellets for 7 days prior to analysis. Error bars show mean +/- SEM. Findings were not significant across groups, as measured by one-way ANOVA.

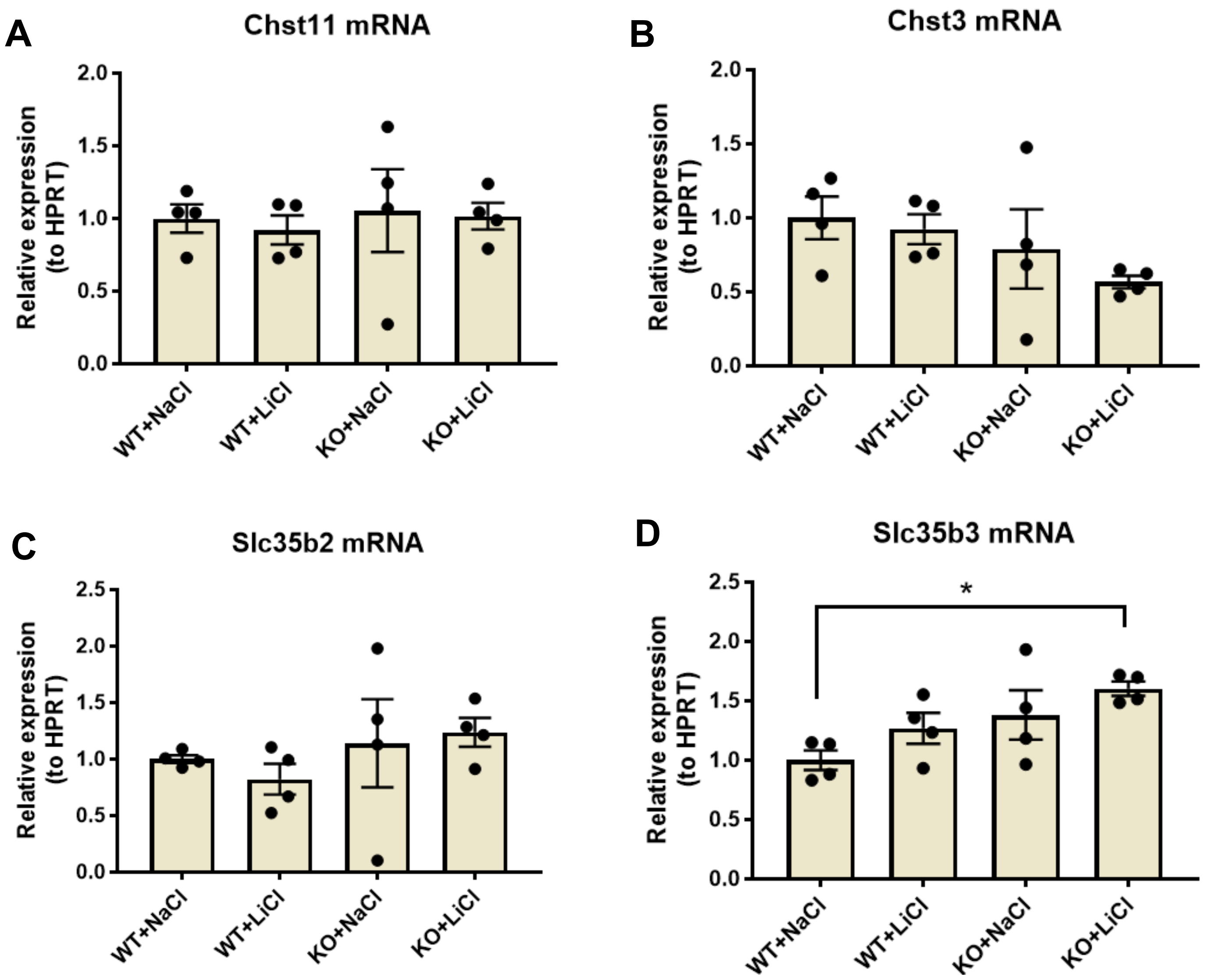

Figure S-2. Gene expression analysis of members of the Golgi-localized sulfation pathway in WT and BPNT2-KO cells, with and without lithium treatment. mRNA expression analysis as measured by quantitative PCR. *Chst11* (A) encodes chondroitin-4-sulfotransferase, *Chst3* (B) encodes chondroitin-6-sulfotransferase, *Slc35b2* (C) encodes PAPST1, *Slc35b3* (D) encodes PAPST2. Expression levels are relative to HPRT expression. Error bars show mean +/- SEM. Findings were not significant across groups, as measured by one-way ANOVA, with the exception of the increase in Slc35b3 expression in KO+LiCl cells relative to WT+NaCl cells (*p=0.0325).

Primer	Sequence
HPRT.Fwd	5'- GCAGTACAGCCCCAAAATGG-3'
HPRT.Rev	5'- ATCCAACAAAGTCTGGCCTGT-3'
BPNT2.Fwd	5'-CGCCGATGATAAGATGACCAG-3'
BPNT2.Rev	5'-GCATCCACATGTTCCTCAGTA-3'
Chst11.Fwd	5'-AAGTATGTTGCACCCAGTCAT-3'
Chst11.Rev	5'-ATGGCAGTGTTGGATAGCTC-3'
Chst3.Fwd	5'-TTCCTGGCATTTGTGGTCA-3'
Chst3.Rev	5'-AGATGCATTCTCCGATAAGAGC-3'
Slc35b2.Fwd	5'-AGGTCCTGAAGCTGGTCTT-3'
Slc35b2.Rev	5'-AATGCTCTCCTGGTGATGTG-3'
Slc35b3.Fwd	5'-CCTTCCTGTTTTCCCTCACTG-3'
Slc35b3.Rev	5'-CATTGCTTTCCTTCCTGTTGTC-3'

Table S1. Primer sequences. Sequences of primers used for quantitative PCR analyses.
